# A review of deep learning-based deformable medical image registration

**DOI:** 10.3389/fonc.2022.1047215

**Published:** 2022-12-07

**Authors:** Jing Zou, Bingchen Gao, Youyi Song, Jing Qin

**Affiliations:** Hong Kong Polytechnic University, Hong Kong, Hong Kong SAR, China

**Keywords:** deformable image registration, medical imaging, clinical applications, deep learning, computer assisted surgery

## Abstract

The alignment of images through deformable image registration is vital to clinical applications (e.g., atlas creation, image fusion, and tumor targeting in image-guided navigation systems) and is still a challenging problem. Recent progress in the field of deep learning has significantly advanced the performance of medical image registration. In this review, we present a comprehensive survey on deep learning-based deformable medical image registration methods. These methods are classified into five categories: Deep Iterative Methods, Supervised Methods, Unsupervised Methods, Weakly Supervised Methods, and Latest Methods. A detailed review of each category is provided with discussions about contributions, tasks, and inadequacies. We also provide statistical analysis for the selected papers from the point of view of image modality, the region of interest (ROI), evaluation metrics, and method categories. In addition, we summarize 33 publicly available datasets that are used for benchmarking the registration algorithms. Finally, the remaining challenges, future directions, and potential trends are discussed in our review.

## 1 Introduction

Image registration, also called image alignment, is a process of establishing spatial transformations between images (1). Image registration has wide applications in various medical image analysis and computer-assisted intervention tasks. According to the type of the transformation, it can be categorized as rigid, affine, and deformable registration (2). A rigid transformation consists of rotation and translation; an affine transformation includes translations, rotations, scaling, and sheering; the two kinds of transformations are described as a 2D single matrix. Unlike rigid and affine transformation, deformable transformation is a high-dimension problem that we need to formulate by a 3D matrix (for 2D deformable registration) or a 4D matrix (for 3D registration), i.e., deformation field. While rigid and affine registration algorithms have already achieved satisfactory performance in many applications, deformable registration is still a challenging task due to its intrinsic complexity, particularly when the deformation is large.

In clinical practice, however, deformable registration can find much more applications than rigid and affine registration. It is utilized to fuse the information that is acquired from different modalities as medical images from different modalities keep different characteristics; for example, magnetic resonance imaging (MRI) provides better contrast for soft tissues, and computed tomography (CT) has clear details of bone (3); fusing these data helps to assure better diagnosis and treatment. Even the images from one modality have distinctions when they are collected at different time points, from different views, or from various people. In this scenario, image registration is used to monitor organ or lesion variation (4).

Additionally, deformable image registration has also been utilized for various computer-assisted interventions in recent years (5–9). For transrectal ultrasound-guided (TRUS) prostate biopsy, it is the most effective way to diagnose prostate cancer, with the advantages of real-time detection, simplicity, and low cost, but for systematic sextant biopsies, its poor imaging quality and lack of sharp contrast between cancer and normal tissue results in false-negative rates of up to 30% (10). Magnetic resonance imaging (MRI), different from TRUS, is the most effective imaging technique for examining anatomical features and targeting prostate tumors. Thus, the deformable registration of pre-operative MR images and inter-operative TRUS images is utilized to fuse their information for enhancing biopsy accuracy. Moreover, registration is also of great significance in radiotherapy (11, 12); it is performed to calculate the offset of the current target from the planned position, and the offset determined from the registration is used to adjust the patient position or the radiation beam.

Deformable image registration aims to calculate the voxel-to-voxel correspondences between a moving image (i.e., source image that needs to be transformed) and a fixed image (i.e., target image used as the template). During image registration, The moving image is transformed to align with the fixed image by minimizing the dissimilarity between the fixed image and the transformed moving image.

Given the source and the target image: *I_S_*, *l*_*T*_∈R^H×W×C^ , the formula is as follows:


(1) $$\underset{f\in F}{\arg\;\min\;}\ell (I_{T},T(I_{S}|{\text{Φ}}_{f}))+\lambda R\left({\text{Φ}}_{f}\right)$$

where *F* represents the function space of *f*, Φ*_f_* denotes Φ with *f* when the input is (*I_S_*, *I_T_*), and ℓ is the loss function to compute the discrepancy between the target image *I_T_* and the registration result *T*(*I_S_* |Φ*_f_*). Additionally, *R*(Φ*_f_*) is the regularization term and the hyperparameter λ is used to balance its importance on the training.

Various traditional registration methods and toolboxes have been devised over the last few decades, e.g., Elastix (13) and ANTs (14). The traditional registration algorithms are constructed as continuous optimization problems (15, 16) or discrete optimization problems (17). Their computational performance, however, is hampered by the high dimension and non-convex properties, and their capability to capture complex deformations is limited (18). Recently, deep learning-based deformable registration methods have greatly attracted researchers’ attention, as data-driven methods benefit significantly from a large number of paired/unpaired images when compared with traditional methods.

Deep learning-based models are capable of improving the deformable registration performance. Firstly, deep neural networks can prompt the iterative optimization procedure to the training stage and achieve fast inference in the test stage. Secondly, neural networks are able to work as an approximator of the similarity between the image pairs to help registration. Thirdly, the deformation field can be predicted directly through an end-to-end model without pre-defining a transformation model.

Though previous reviews about deep learning-based medical image registration literature have been published (19–22), there are still some deficiencies. On one hand, these reviews lack a summary of the public dataset for benchmarking registration algorithms. On the other hand, some details of the selected literature were missing. This review will serve as a supplement to them by adding more recent studies, by discussing the selected literature with comprehensive details, by concluding the most commonly used public dataset, and by providing some suggestions about remaining problems and further research.

Specifically, our review aims to

1. Conclude the most commonly used public datasets with details on modality, organ, dimension, quantity, disease, release time, etc.
2. Summarize literatures on deep learning-based deformable image registration, especially for recent research, and list organ, modality, dimension, model, evaluation metrics, publication source, etc. in tables.
3. Provide detailed statics on research interests (Modalities, ROIs, Evaluation metrics, and Methods).
4. Discuss the remaining issues that need to be studied and the directions for future research.

Other contents of this review are organized as follows: In *Section 2*, we present detailed statistics on modalities, organs, etc. *Section 3* summarizes the frequently used dataset. We discuss deep learning-based deformable medical registration methods from five categories in *Section 4*. Then, we discuss the limitations and future potential in *Section 5*. *Section 6* concludes the review.

## 2 Statistical analysis

**Figure 1:**
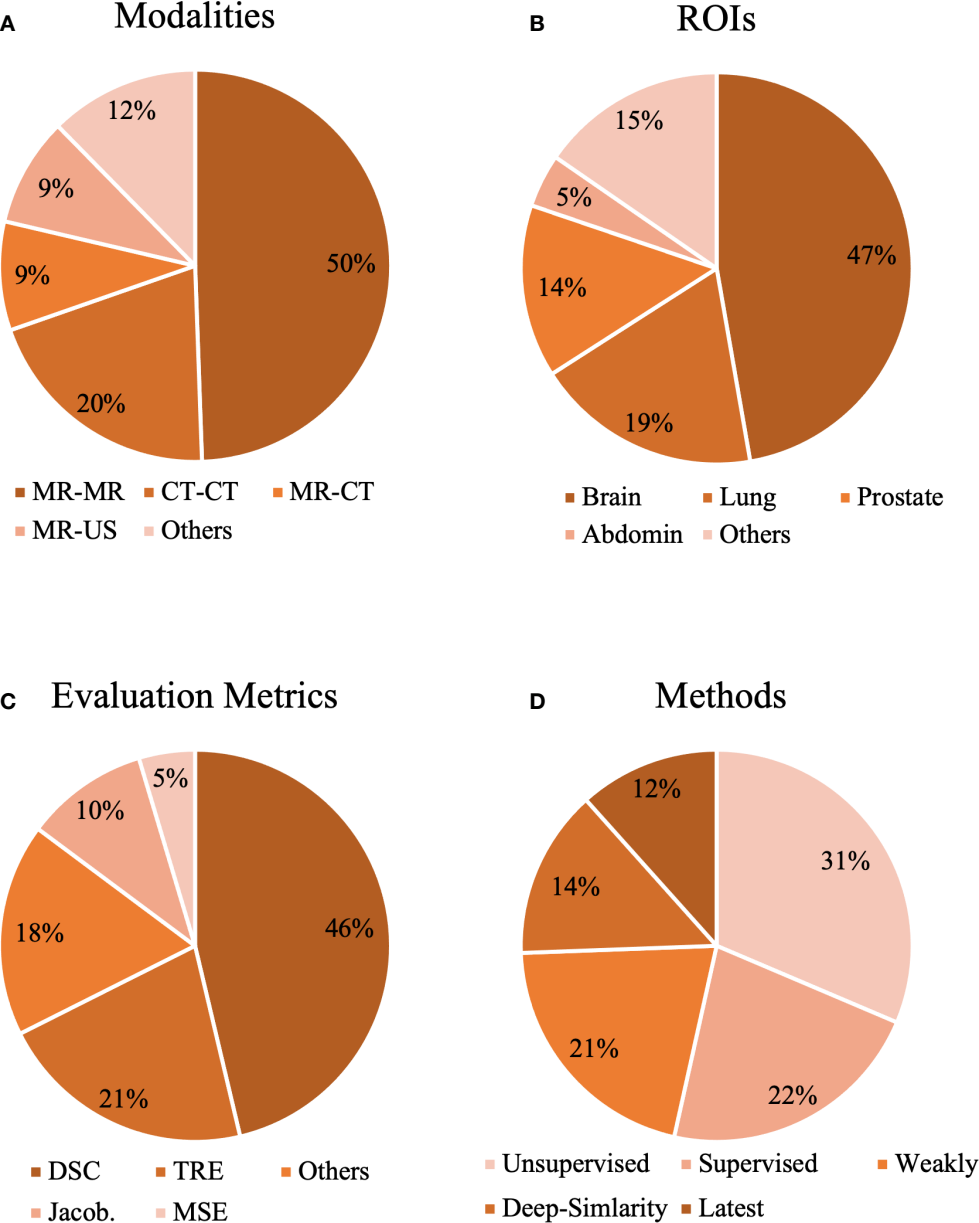
The statistical analysis of our selected papers from the aspects of **(A)** modalities, **(B)** organs, **(C)** evaluation metrics, and **(D)** methods.

For the purpose of this review, as completely as possible, to include relevant studies in the past decade and potentially advanced studies in the upcoming decade, this review mainly includes “deep learning”, “medical image registration”, “supervised”, “unsupervised”, “motion estimation”, “GAN”, “deep similarity”, “Transformer”, “contrastive learning”, “meta learning” and “knowledge distillation” as the search keywords. Due to the fact that medical image registration and deep learning-based methods could be involved in different conferences and journeys focusing on various specializations, the conference and journal sources of the selected papers were from but were not limited to Computer Vision and Pattern Recognition (CVPR), Medical Image Computing and Computer Assisted Interventions (MICCAI), Social Science and Management Innovation (SSMI), Medical Image Analysis (MIA), Innovative Management, Information Production (IMIP), Technology Modernization and Innovation (TMI), International Symposium on Biomedical Imaging (ISBI), Machine Learning Machine Intelligence (MLMI), and Medical Imaging With Deep Learning (MIDL). Google Scholar, arXiv, and PubMed were searched to find the targeted publications. Papers that do not notify the details about the training datasets such as not clarifying the name, the number of participants, and implementation descriptions were excluded in this review. Other papers that do not clearly state the methods and validations were also excluded in this review. After the selection and screening, 91 studies related to learning-based medical image registration were finally included. **Figure 1** describes the statistical results of the studies in this review.

Registration in mono-modality (MR-MR, CT-CT, etc.) and multimodality (MR-CT, MR-US, etc.) can be seen in **Figure 1A**. As shown in **Figure 1A**, MR-MR and CT-CT are the most commonly studied modalities, and each accounts for 50% and 30% of the selected studies, which might indicate that registration in mono-modality is relatively convenient and simple to implement and registration in cross-modalities encounter more challenges. Based on ROIs, we collected methods for the brain, lung, prostate, heart, liver, knee, torso, and abdomen in **Figure 1B**. The three most popular ROIs being investigated are brain, lung, and the prostate, and each accounts for 47%, 19%, and 14% of all the studies. The public datasets normally contain scans within these three ROIs as common diseases exist in these regions. Thus, studies utilizing public datasets could be more inclined to investigate the brain, lung, and prostate. With respect to evaluation metrics, cross-correlation (CC), mean square error (MSE), Dice coefficient (DSC), target registration error (TRE), Jacobian determinant (Jaco.Det.), and other metrics are shown in **Figure 1C**. The evaluation metrics such as the MSE measure the numerical difference between warped images and reference images, and other metrics such as the Jacobian determinant quantifies the smoothness of the deformation field to keep the results of the registration plausible. Based on **Figure 1C**, DSC is the most popular metric and accounts for 46% of all the studies. DSC would be used if the ground truth labels were provided, and this statistical analysis demonstrates the current trend for validating the accuracy of the proposed registration model that commonly requires the segmentation labels as the ground truth. This review also demonstrates the ratio of similarity-based, supervised, weakly supervised, unsupervised, and latest methods in **Figure 1D**. Unsupervised and supervised methods are two most popular methods and each accounts for 31% and 22% of all the studies. Although the ratio of latest methods is the smallest, this review tends to provide certain insights and suggest potential trends in developing deep learning methods of medical image registration.

## 3 Datasets

The absence of relevant data, as one of the main bottlenecks in current learning-based medical image analysis, resulted from several challenges including data collection with limited but subtle equipment, label annotation with human expertise, and data access under ethical considerations. Compared to commonly used datasets such as the MINST (23), qualified medical images cannot be collected from handwritten ones but need to be acquired by strict protocols and expensive scanning devices. Considering MRI method as the example, each session generally lasts 2 h under the supervision and manipulation of professional researchers, and during the scanning process, the subjects are almost fully constrained in the specialized machine in order to obtain accurate data (24). Other than sophisticated protocols to collect data mentioned above, labels and the annotation of the medical images have to be achieved by specialized professionals. Unlike datasets such as the autopilot that could be labeled by less professional annotators, in order to keep the accuracy and possibly avoid the bias of the ground truth, a single scan might be labeled by multiple experts. Finally, accessing medical data is not allowed for most researchers since the clinical data contain private information and only researchers with authorization could utilize the dataset (25).

Overall, based on the characteristics of the medical dataset discussed above, prevalent and testified public databases are thus normally chosen by research teams in deep learning-based medical image analysis. Moreover, in addition to alleviating the challenges mentioned, the training and assessment of DL models on multiple public datasets could also better demonstrate the generalizability of the proposed methods and clearly compare the results on fair benchmarks, including computational time and registration accuracy. Our review summarized 30 public datasets from deep learning registration studies in **Table 1**. Most of the datasets consist of brain MR and lung CT images, and other modalities in different ROIs such as the abdomen and knee were also included. The ADNI dataset as one example in brain datasets contains MRI longitudinal image scans in AD patients from 63 sites across the US and Canada. ADNI develops standardized protocols for multi-center comparison and helps over 1,000 scientific publications explore the prevention and treatment of AD since 2004 and would be funded further in the future (26). COPDGene as one example in lung datasets contains chest CT scans of more than 10,000 individuals for chronic obstructive pulmonary disease (COPD). More than 375 publications have used COPDGene to explore the assessment and identify the biomarkers of COPD since 2009 (47). OAI as the example in the knee dataset contains MRI and x-ray longitudinal measurements from 4,796 subjects, and more than 400 research manuscripts have used the OAI to explore the assessments and interventions of knee osteoarthritis (48). PROMISE12 as the example in the prostate dataset is the dataset made for MICCAI prostate segmentation challenge, which was used by 11 academic teams for various segmentation algorithms and showed promising results (55).

**Table 1:** Public dataset for benchmarking medical image registration.

Name	Modality	Organ	Dimension	Quantity	Time	Disease	Remark
ADNI (26)	MRI, fMRI	Brain	3D	819	2004-2021	AD/MCI	Longitudinal changes in AD/MCI
OASIS (27)	MRI, PET	Brain	3D	1664	Since 2007	AD	Longitudinal study in AD
ABIDE (28)	MRI	Brain	3D	1112	2014	ASD	20 samples from 17 sites
ADHD20 (29)	MRI, RS-fMRIn	Brain	3D	776	2012	ADHD	An fMRI dataset for ADHD
MCIC (30)	MRI, fMRI, DWI	Brain	3D	161	2013	Schizophrenia	Multimodal imaging for schizophrenia.
PPMI (31)	MRI	Brain	3D	423	2011	PD	Identify biomarkers of PD progression
HABS (32)	MRI, fMRI, PET	Brain	3D	284	2015	Normal	MRI and PET acquisitions
GSP (33)	MRI, fMRI	Brain	3D	1570	2015	Normal	Rapid imaging protocol
LPBA40 (34)	MRI	Brain	3D	40	2007	Normal	56 labeled structures
IBSR18 (34)	MRI	Brain	3D	18	2009	Normal	84 labeled regions
CUMC12 (34)	MRI	Brain	3D	12	2009	Normal	128 labeled regions
MGH10 (34)	MRI	Brain	3D	10	2009	Normal	72 labeled regions
MindBoggle 101 (35)	MRI	Brain	3D	101	2012	Normal	Labeled cortices with DKT protocol
Freesurfer buckner40 (36)	MRI	Brain	3D	40	2012	Normal	Dataset in Freesurfer software
BraTS (37)	MRI	Brain	3D	More than 542	Since 2012	Tumor	Tumor segmentation
ALBERTs (38)	MRI	Brain	3D	20	2012	Normal	Cerebral MRIs of newborns
Simulated Brain (39)	MRI	Brain	3D	20	2006	Normal	Simulated medical imaging
RESECT (40)	MRI, US	Brain	3D	23	2017	Glioma	Registration for brain tumor resection.
IXI (41)	MRI	Brain	3D	662	2014	Normal	Include T1, T2, PD, MRA and DTI images
DirLAB (42)	CT	Lung	3D	10	2009	Normal	300 landmarks
POPI (43)	CBCT	Lung	3D	6	2011	Normal	100 landmarks
SPREAD (44)	CT	Lung	Unknown	144	2007	COPD	Longitudinal design in 30 months
TCIA (45)	CBCT, CT	Lung	3D	More than	2017	Lung Cancer	Database for cancer research
EMPIRE10 (46)	CBCT	Lung	3D	30	2010	Normal	Intra-patient registration
COPDgene (47)	CT	Lung	3D	More than 10,000	2011	COPD	Investigate COPD
OAI (48)	MRI	Knee	3D	4796	Since 2003	Osteoarthritis	Osteoarthritis dataset
KiTS19 (49)	CT	Kidney	3D	300	2019	Kidney tumor	Partial or radical nephrectomy patients
MSD (50)	MRI, CT	Whole Body	3D	2,633	2019	Multiple disease	Whole body
VISCERAL (51)	CT, MRI	Whole Body	3D	More than 120	Since 2016	Unknown	Whole body
Pancreas-CT (52)	CT	Pancreas	3D	82	2015	Unknown	Expert labeled CT volumes
SpineWeb (53)	MRI, CT	Spine	3D	Multiple in 16 datasets	Since 2012	Spinal injury	Detect vertebral body fractures
Prostate-3T (54)	MRI	Prostate	3D	64	2013	Unknown	Segmentations of central gland and the peripheral zone
PROMISE12 (55)	MRI	Prostate	3D	100	2013	Unknown	Prostate MRI dataset

AD, Alzheimer disease; MCI, mild cognitive disease; ASD, autistic spectrum disorders; ADHD, attention deficit hyperactivity disorder; PD, Parkinson disease; COPD, chronic obstructive pulmonary disease; DWI, diffusion-weighted imaging; MRI, magnetic resonance imaging; PET, positron emission tomography; CBCT, cone-beam computed tomography; DTI, diffusion tensor imaging; fMRI, functional magnetic resonance imaging.

In **Table 1**, the date of publication for each dataset was also listed. Some datasets are still collecting data for further imaging analysis support, such as the data collected for BraTs since 2012 (37). The quantity of each dataset demonstrates the size of each dataset, and the remarks of each dataset illustrate the significance of each dataset.

## 4 Methods

**Figure 2:**
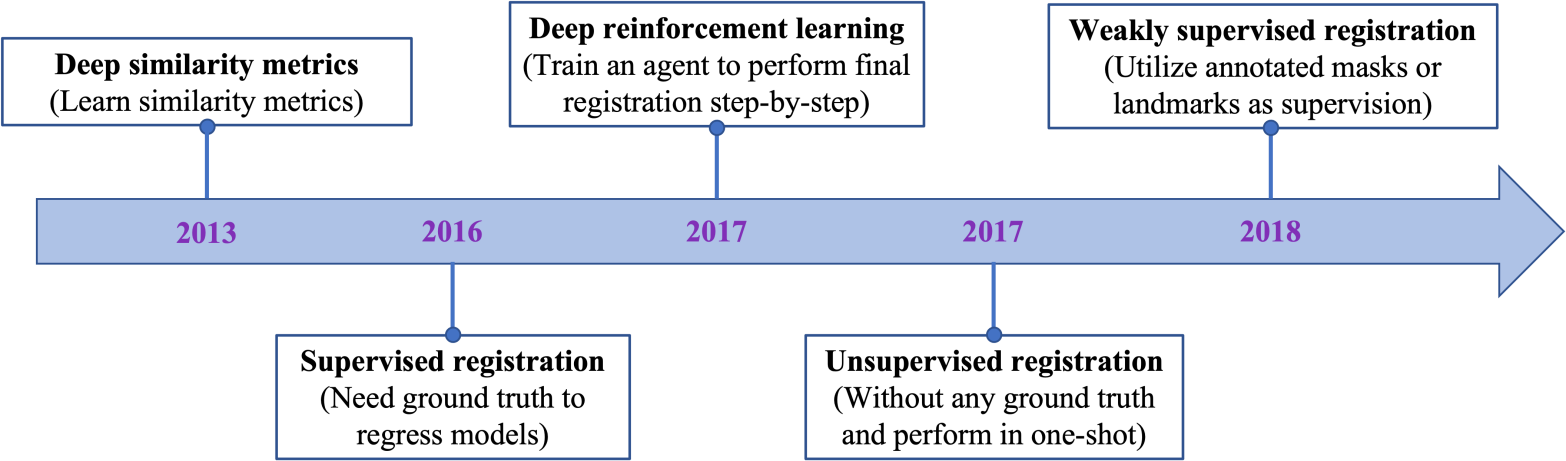
The evolution of deep learning-based methods on deformable medical registration from deep similarity methods to weakly supervised methods.

**Figure 2** displays the chronological development of deep learning-based deformable medical image registration. In this review, deep learning-based registration methods are classified into the following four categories by evolution: Deep Iterative Methods, Supervised Methods, Unsupervised Methods, and Weakly Supervised Methods.

### 4.1 Deep iterative methods

Early research on deep learning-based registration directly extended the traditional registration framework by using deep neural networks as an approximator of the similarity or dissimilarity between the source image and the target image. Another early research direction is reinforcement learning. This paradigm trained an agent to perform a sequence of actions and iteratively improve image alignments by maximizing rewards. These two kinds of paradigms both adopt iterative strategy.

#### 4.1.1 Deep similarity metrics

For traditional image registration methods, the commonly used similarity metrics are intensity-based, including mean square distance (MSD), sum-of-square distance (SSD), (normalized) mutual information (MI), and (normalized) cross-correlation (CC). Generally, these intensity-based similarity measurements work quite well for mono-modality image registration (e.g., CT-CT and MRI-MRI image registration), in which the image pair shares similar intensity distribution.

**Figure 3:**
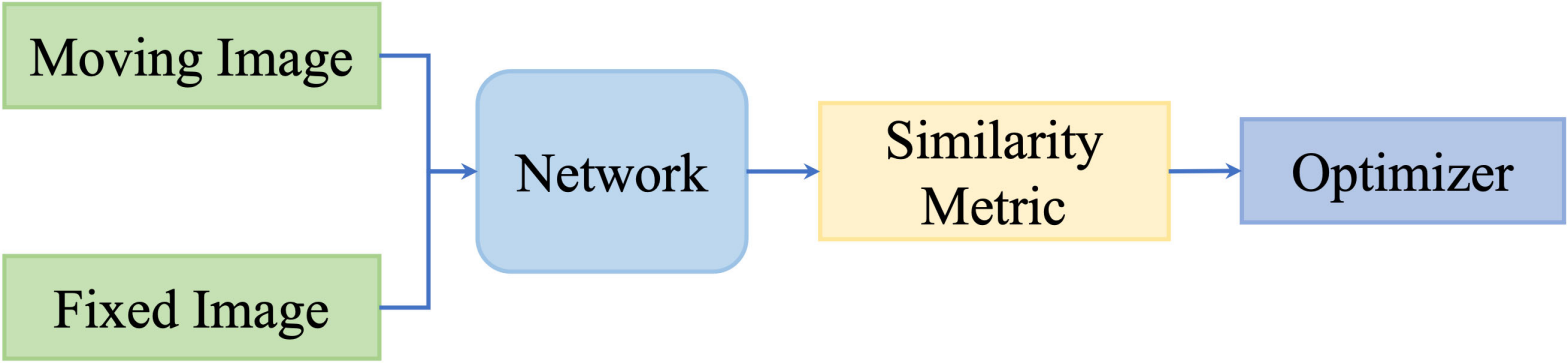
Illustration of deep similarity metric-based registration methods.

However, these metrics focus on intensity values; thus, they are not capable of measuring multimodality registration due to the diverse intensity distributions across modalities. To take advantage of deep neural networks, researchers proposed to replace the intensity-based similarity metrics with metrics that have been learned through deep networks and that achieved promising registration performance. **Figure 3** shows the general pipeline of deep similarity-based registration methods.

Wu et al. (56) firstly proposed to adopt deep learning technology to obtain the similarity metric for registration. They adopted convolutional-stacked autoencoder (CAE) to extract the discriminative features for 3D deformable brain MRI registration. Then, the registration was performed by optimizing the NCC of the two features and improved registration accuracy was achieved. So et al. (57) proposed a novel learning-based metric by using Bhattacharyya Distances. The dissimilarity of the testing image pairs is calculated by incorporating the expected intensity distributions learned from the registered training image pairs. A list of research of this category is presented in **Table 2**.

**Table 2:** Medical image registration methods based on deep similarity metrics.

Reference	Organ	Modality	Dimension	Model	Evaluation	Source
(56)	Brain	MRI	3D	SAE	DSC	MICCAI 2013
(58)	Lung, Brain	Synthetic	2D, 3D	CNN	Convergence time	CMMM 2015
(59)	Brain	MRI	3D	SAE	DSC	TBE 2015
(60)	Brain	T2–T1	3D	CNN	DSC	MICCAI 2016
(61)	Brain	Pathological	2D	SAE	Deformation error	SSMI 2016
(62)	Brain	MRI	3D	CNN	SSIM, PSNR	PRL 2017
(57)	Brain	MRI	2D, 3D	CNN	TRE	PR 2017
(63)	Brain	T1–T2, T1–PD	3D	CNN	TRE	Sensors 2018
(64)	Brain, Abdomen	MRI, CT	3D	CNN	DSC	JBHI 2018
(65)	Lung	CT	3D	CNN	TRE	JMI 2018
(66)	Brain	MRI	3D	CNN	TRE	MBEC 2019
(67)	Brain	MRI	3D	CNN	TRE, DSC	MIA 2021

MRI, magnetic resonance imaging; CT, computed tomography; SAE, stacked autoencoder; CNN, convolutional neural network; DSC, Dice coefficient; SSIM, structural similarity index measure; PSNR, peak signal-to-noise ratio; TRE, target registration error.

So far, a number of registration methods for medical image based on deep similarity metrics have been studied and have shown great potential in multimodality image registration. However, a sufficient number of explicitly annotated ground truths are required for deep similarity metric training, which hinders the development of such approaches. Moreover, it is difficult to interpret the learned deep similarity metrics, and the iterative process still limits the use of these methods. Nowadays, the number of literatures for this category has decreased, and this trend is projected to continue.

#### 4.1.2 Deep reinforcement learning

**Figure 4:**
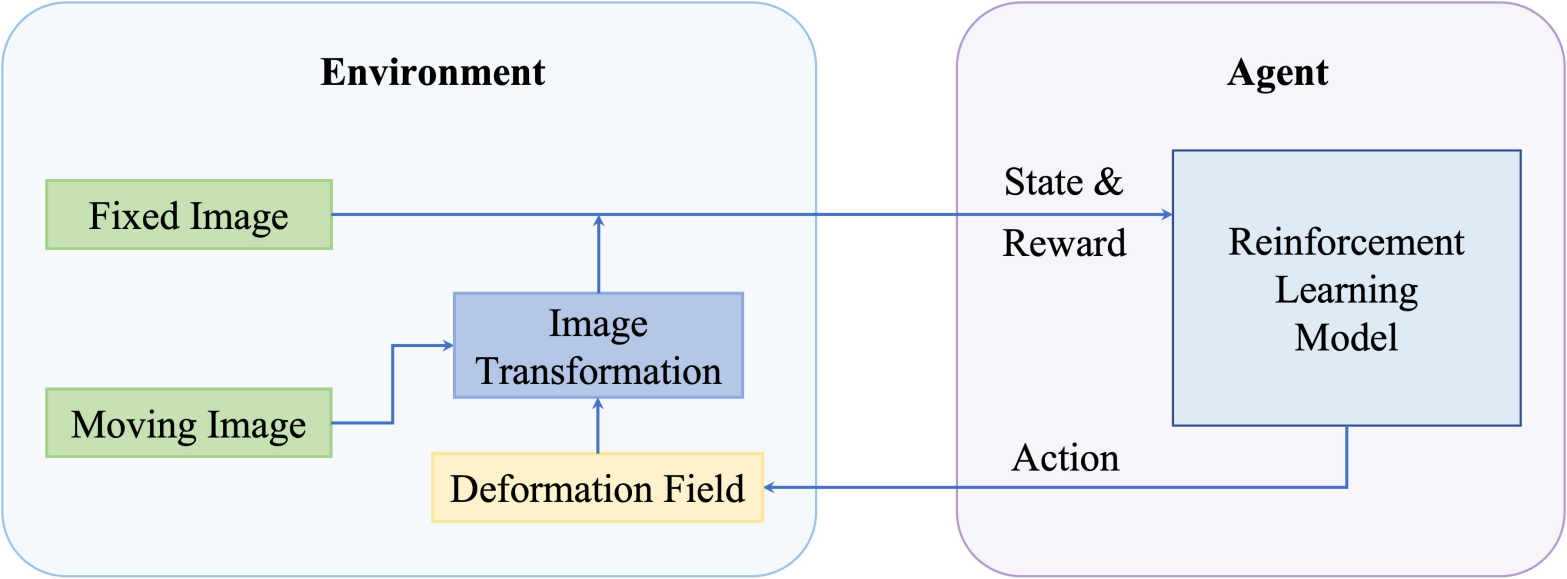
A general pipeline of deep reinforcement learning based deformable image registration methods.

Since the initial publishing from Mnih et al. (68) and Silver et al. (69), Reinforcement Learning (RL) has gained a lot of attention and has been used for diverse applications including robotics, video games, and healthcare. In RL, an intelligent agent learns to map states to actions iteratively to maximize rewards received from a designed environment. For RL-based registration, the inputted image pairs are constructed in a given environment, and an artificial agent learns to generate the final transformation subsequently so that the rewards received from that environment can be maximized. A general pipeline of deep reinforcement learning-based deformable image registration methods is illustrated in **Figure 4**.

Liao et al. (70) firstly proposed to train an artificial agent for the rigid registration of 3D CT images and cone-beam CT (CBCT) images for cardiac and abdominal images. Ma et al. (71) adopted deep reinforcement learning to extract the best feature representation to minimize the discrepancy between CT and MRI images for rigid registration. Instead, there are a few studies about RL-based algorithms on deformable registration. In 2017, Krebs et al. (72) applied RL to deformable prostate MRI registration by training an agent to investigate the parametric space of a statistical deformation model constructed with training data.

Different from deep similarity metrics-based methods, the similarity measures in these kinds of methods are routinely provided in a traditional way, e.g., MSE, NMI, or LCC. Thus, they have limited applications in multimodality registration. Moreover, RL-based methods mainly focus on rigid registration, as it is hard for agents to tackle the large state space generated from deformable vector field.

### 4.2 Fully supervised methods

**Figure 5:**
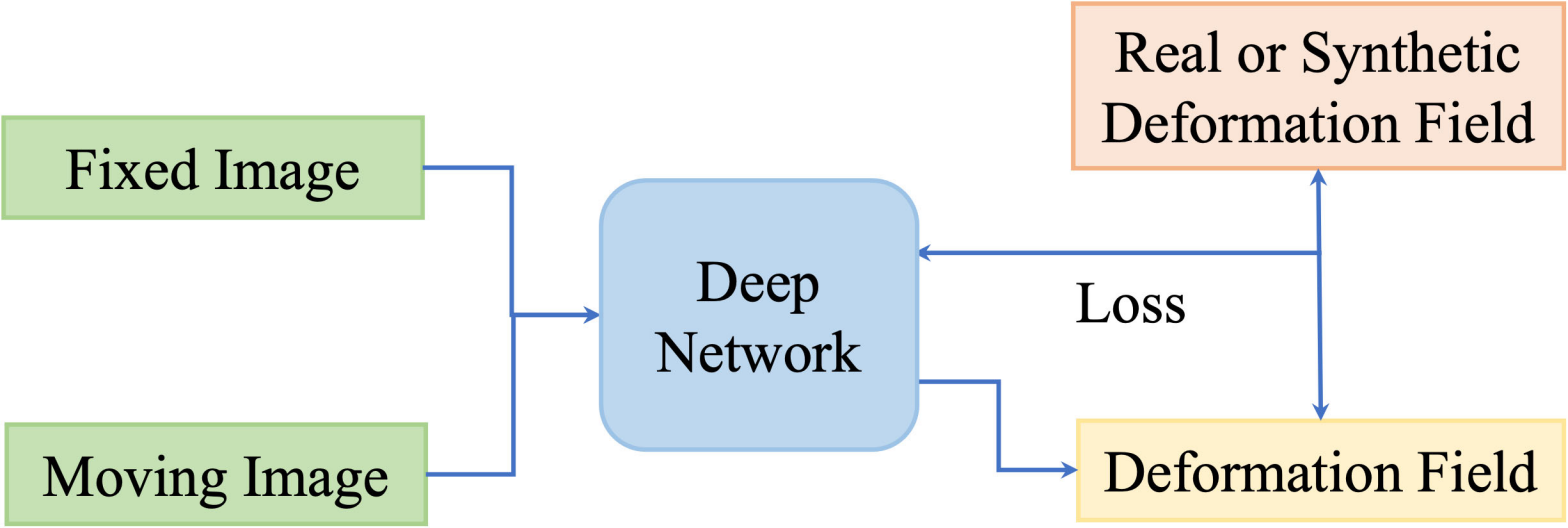
Illustration of fully supervised registration methods.

One disadvantage of deep iterative methods is that the registration process is time-consuming and iterative. Fully supervised methods help to predict transformations in one shot by the supervision of real deformation vector fields or synthetic deformation fields. The real deformation fields are generated from traditional registration, and the synthetic deformation fields are obtained from statistical models or random transformations. **Figure 5** illustrates the general framework of the fully supervised methods.

At first, Yang et al. (73) proposed to register brain MRI images using a U-Net-like structure in one step. The large diffeomorphic metric mapping was utilized to generate a basis, then the original momentum values of the pixels from the images are inputted into the network, and the values are refined to predict the deformation field. Fan et al. (74) presented a Birnet for brain MRI image registration by utilizing the deformation field estimated from the traditional registration method. They also proposed gap-filling to learn more high-level characteristics and designed multi-channel inputs to learn more information. Recently, Fu et al. (75) designed an MR-TRUS registration network for prostate interventions; the supervision deformation field is generated from population-based FE models from point clouds with biomechanical constraints.

These methods have achieved notable results with real deformation fields as supervision. However, supervision by real deformation fields is limited by the size and the diversity of the dataset. Then, synthetic deformation fields are developed for the learning of deformation fields.

Rohe et al. (76) adopted a U-Net-like network to predict the deformation field for 3D cardiac MRI volume registration. The supervisions are transformations generated from mesh segmentations, and SSD between the supervision and prediction is set as the loss function. Their results are comparable with those from traditional registration. Sokooti et al. (77) presented a multi-scale network to learn a deformation field of intra-subject 3D chest CT registration. They used random DVF as supervision. Uzunova et al. (78) designed a network for the registration between 2D brain MRI and 2D cardiac MRI. Their ground truth is generated utilizing statistical appearance models (SAMs). They adapted FlowNet (79) architecture and obtained outperforming results.

The spatial transformer networks (STNs) (80) introduced in 2015 is one significant advancement that is beneficial in this era. STN is composed of three parts. The first is a localization network, whose goal is to use the input features to regress the transformation parameters. The second part is a grid generator, which generates a sampling grid that will be used to sample the input feature map. Another is a sampler that will produce the transformed feature map from the sampling grid and the input feature map by sampling and interpolation. STN can be inserted anywhere in various networks to execute a spatial transform on an input feature map because it is a completely differentiable module.

The fully supervised methods are widely studied and have notable results. A list of works about these methods are presented in **Table 3**. However, the generation of real or synthetic deformation fields is hard, and these deformation fields are different from the real ground truth, which will confine the accuracy and efficiency of these kinds of methods. In this situation, unsupervised methods are promising to tackle the problems.

**Table 3:** Fully supervised medical image registration methods.

Reference	Supervision	Organ	Modality	Dimension	Model	Evaluation	Source
(73)	Real DVF	Brain	MRI	2D, 3D	CNN	Deformation error, [*J*]	DLMIA 2016
(81)	Real DVF	Brain	T1–T2	3D	CNN	Deformation error, [*J*]	ISBI 2017
(82)	Real DVF	Brain	MRI	3D	CNN	Deformation error, [*J*]	NeuroImage 2017
(83)	Registered data	Drosophila	TEM	2D	CAE	DSC	DLMIA 2017
(77)	Synthetic DVF	Lung	CT	3D	CNN	MAE, TRE	MICCAI 2017
(84)	Synthetic DVF	Brain	MRI	3D	CNN	DSC, ASSD	MICCAI 2017
(76)	Synthetic DVF	Cardiac	MRI	3D	CNN	DSC, HD, [*J*]	MICCAI 2017
(85)	Synthetic DVF	Craniofacial	X-ray-CBCT	2D-3D	CNN	Intensity distance	DLMIA 2017
(78)	Synthetic DVF	Brain	MRI, Cardiac	D	CNN	Jaccard	MICCAI 2017
(86)	Similarity	Prostate	CT-MRI	3D	CNN	DSC, ASD	MLMI 2018
(87)	Real DVF	Lung	CT	3D	CNN	TRE	IAMO 2018
(88)	Synthetic DVF	Lung	CT	3D	CNN	TRE	TMI 2018
(89)	Real DVF	Abdomen	MRI	3D	CNN	SNR	BJR 2018
(74)	Real DVF	Brain	MRI	3D	FCN	DSC	MIA 2019
(90)	Synthetic DVF	Lung	CT	3D	CNN	TRE, folding, [*J*]	arXiv 2019
(91)	Synthetic DVF	Prostate	MRI-US	3D	CNN	SRE	CMIG 2020
(92)	Real DVF	Head	MRI-CBCT	3D	CNN	DSC, MSD, HD	MI 2020
(93)	Real DVF	Lung	CT	3D	CNN	MSE, SSIM	QIMS 2021
(75)	Real DVF	Prostate	MRI-US	3D	CNN	DSC, MSD, HD	MIA 2021

DVF, deformation vector field; MRI, magnetic resonance imaging; TEM, transmission electron microscope; CBCT, cone-beam computed tomography; US, ultrasound; FCN, fully convolution network; [J], Jacobian determinant; ASSD, average symmetric surface distance; CAE, convolution autoencoder; TRE, target registration error; ASD, average surface distance; SNR, signal-to-noise ratio; SRE, surface registration error; SSIM, structural similarity index measure; MSD, mean surface distance; MSE, mean square error; HD, Hausdorff distance.

### 4.3 Unsupervised methods

Even though different data augmentation techniques and data collection methods have been utilized in supervised learning, the preparation of the required ground truth is inconvenient and leads to the fact that the supervised framework has limitations in generalizing results in different domains and applying various registration tasks. Thus, the unsupervised registration has a more convenient training process that usually only inputs paired images without predefined DVF as the ground truth. The formulation in the loss function of the learning network constrains the output DVF to be accurate. Generally, the unsupervised learning framework consists of the similarity-based methods and GAN-based methods. Unsupervised methods are concluded in **Table 4**.

**Table 4:** Unsupervised medical image registration methods.

Reference	Loss	Organ	Modality	Dimension	Model	Evaluation	Source
(86)	NCC	Prostate	CT-MRI	3D	CNN	DSC	MLMI 2018
(94)	MSE, LNCC	Brain	MRI	3D	CNN	DSC	CVPR 2018
(95)	MSE, NCC	Brain	MRI	3D	CNN	DSC	MICCAI 2019
(96)	CC	Brain	MRI	3D	CNN	DSC	SASHIMI 2019
(97)	CC	Lung	CT	3D	CNN	TRE	MICCAI 2019
(98)	NCC	Knee	MRI	3D	CNN	TRE,RMSE	CVPR 2019
(99)	NCC	Lung	CT	4D	CNN	TRE	PMB 2020
(100)	MSE	Prostate	MR-Histology	4D	CNN	TRE, Dice	MIA 2021
(101)	CC	Lung, Brain	CT, MRI	3D	CNN	TRE, DSC, [*J*], Time	MIA 2021
(102)	MSE, NCC	Prostate	US-MRI	2D-3D	CNN	DSC, HD	MICCAI 2021
(103)	NCC	Lung	CT	3D	CNN	TRE	MICCAI 2021
(104)	Similarity loss	Brain	T1, T2, FLAIR	3D	CNN	DSC, RMSE	MICCAI 2021
(105)	Similarity loss	Brain	MRI	3D	CNN	DSC, RMSE	MICCAI 2021
(106)	MI	Brain	MRI	3D	CNN	DSC	TMI 2021
(107)	NLCC	Hippocampus, Prostate	MRI	3D	CNN	MSE, NLCC, DSC	WACV 2022
(108)	MSE	Porcine	Endoscopic	3D	CNN	MSE, NLCC, [*J*]	IJCARS 2022
(109)	MSE, LNCC	Brain	MRI	3D	CNN	Dice	IPMI 2021
(110)	MIND	Abdominal	CT-MR	3D	CNN	Dice, ASD	ISBI 2021
(111)	MSE, MIND	Abdominal	CT-MR	3D	CNN	Dice, ASD	MICCAI 2022
(112)	MIND	Abdominal	CT-MR	3D	CNN	Dice, ASD, [*J*]	IJCARS 2021
(113)	cycloss	Brain	MR-CT	2D	cycGAN	MAE, PSNR	SASHIMI 2017
(114)	cycloss	Thorax, Abdomen	MR-CT	3D	cycGAN	NMI, MIND	CVPR 2018
(115)	GAN-loss	Prostate	MR-TRUS	3D	Wasserstein GAN	TRE, D-score	MLMI 2018
(116)	NMI, SSIM, VGG	Retina, Cardiac	Fundus-FA, MRI	3D	cGAN, cycGAN	Dice, HD95, MSE	CVPR 2019
(117)	Local Gradient Loss	Brain	MRI	3D	Deform-GAN	MI, NGF, LCC	CVPR 2020
(118)	cycloss	Cardiac	CT-TEE	2D	cycGAN	DR, HD95, ASD	CMMM 2020
(119)	cycloss	Kidney, Abdomen	CT-MR	3D	DS cycGAN	SSIM, PSNR	MICCAI 2020
(120)	NCC	Brain	MRI	3D	GAN	DSC, HD, ASD, CC	MICCAI 2021
(121)	cycloss	Brain, Aortic	CT-MR	2D, 3D	Dicyc-GAN	MSE, PSNR, SSIM	Info Fusion 2021
(122)	LNCC	Brain	MRI	3D	GAN	Dice, [*J*]	ICCV 2021
(123)	NCC	Head	MRI-CT	3D	cycGAN	Dice, SD, HD, TRE, [*J*], Time	MIA 2022
(124)	NCC	Lung	CT	2D	GAN	Dice, HD, ASSD, [*J*]	CMIG 2021

CT, computed tomography; MRI, magnetic resonance imaging; TEE, transesophageal echocardiography; FA, fluorescein angiography; CNN, convolutional neural network; FLAIR, fast fluid attenuated inversion recovery; TRUS, transrectal ultrasound; DSC, Dice coefficient; GAN, generative adversarial network; ASSD, average symmetric surface distance; TRE, target registration error; CC, cross-correlation; cGAN, conditional generative adversarial network; cycGAN, cycle generative adversarial network; cycloss, consistency loss; HD, Hausdorff distance; ASD, average surface distance; STD, standard deviation; CC, Pearson’s correlation coefficient; RMSE, root mean square error; NMSE, normalized mean square error; SSIM, structural similarity index measure; PSNR, peak signal-to-noise ratio; VGG, L2 distance between two images; MIND, modality independent neighborhood descriptor; NGF, nerve growth factor; [J], Jacobian determinant; MI, mutual information; SD, surface distance; NLCC, normalized local cross-correlation; NLCC, localized normalized cross-correlation.

#### 4.3.1 Similarity-based unsupervised methods

**Figure 6:**
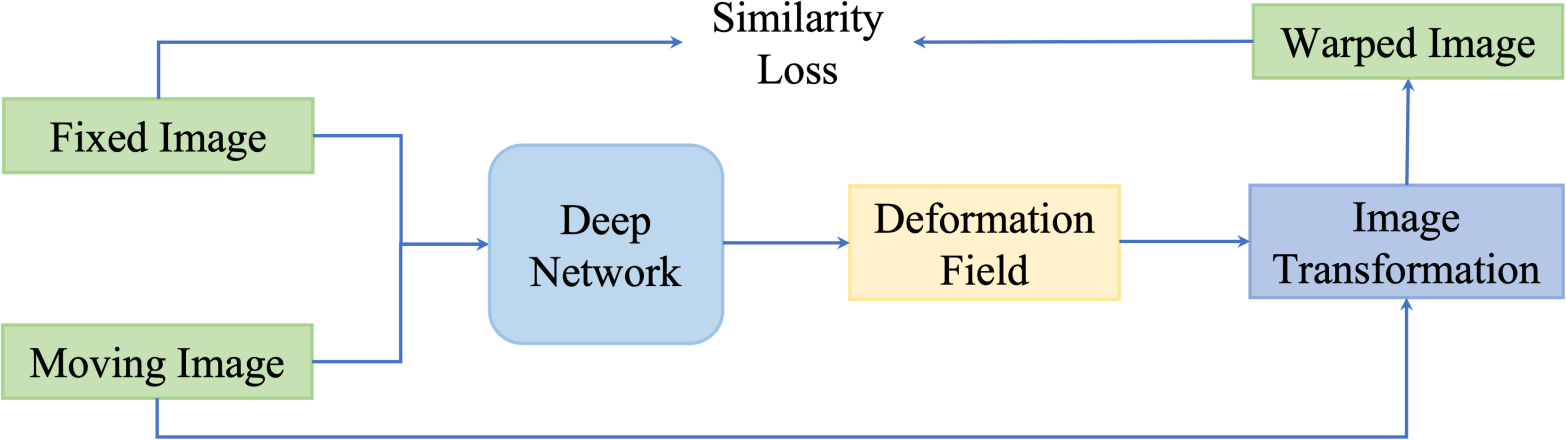
A general pipeline of similarity based deformable image registration methods. This network is optimized by optimizing a similarity loss function.

These kinds of methods update networks by minimizing the dissimilarity between the fixed images and the transformed moving image. An illustration of similarity-based unsupervised methods is presented in **Figure 6**.

Balakrishnan et al. (94) designed a U-Net framework named VoxelMorph to perform DIR of brain MR images. Unlike conventional registration methods that calculate the DVF for every pair of images, they formulate the DVF as a global function that could be optimally parameterized with the trained convolutional neural network. Given the only paired images as the inputs, VoxelMorph rapidly predicts the relevant DVF and uses it to get the wrapped image. The loss function includes an unsupervised setting that minimizes the warped image and fixed image based on image intensity metrics, and an auxiliary supervised setting that minimizes the annotated segmentation errors. The proposed method performed comparably to conventional registration methods in terms of the Dice score. Estienne et al. (125) used a shared encoder with a separate decoder named U-ResNet to compute the DVF. The network inputs paired fixed and moving images and aims to output their specific segmentation maps. The registration accuracy would be optimized based on the Dice score in the segmentation results.

Not only applied in brain MR images, De et al. (126) deployed a similar unsupervised framework in cardiac MRI and chest CT. They combined the unsupervised affine and deformable registration and downsampled the input images into multiple stages, which better captures the small motions and improves the registration results. Shao et al. (127) implemented this similar coarse-to-fine registration strategy in prostate MRI images named ProsRegNet. Shen et al. (98) designed a three-phase unsupervised registration framework to calculate a transformation map for knee MRI images in a longitudinal study.

Recently, Chen et al. (105) extended this registration framework to infant tasks. They proposed an unsupervised age-conditional cerebellum atlas construction framework. Given the age input and two temporally adjacent source images, it would generate a longitudinally consistent 4D infant brain atlas with the longitudinal constraint in the loss function. Kim et al. (101) introduced the CycleMorph, which added the cycle consistency in the loss function to preserve the topology of the predicted DVF. Guo et al. (102) fused 2D TRUS image with 3D MRI volume with the frame-to-volume registration network (FVR-NET). They performed the 2D TRUS image and 3D TRUS volume registration by adopting a dual branch feature extraction module, and used the output transformation parameters to combine with the registration of 3D TRUS and 3D MRI.

Many groups have proved that their proposed similarity-based method could achieve SOTA performance compared to conventional methods; however, they mainly focus on mono-modality and the image intensity similarity metrics would be inappropriate in multimodality registration tasks.

#### 4.3.2 GAN-based unsupervised methods

**Figure 7:**
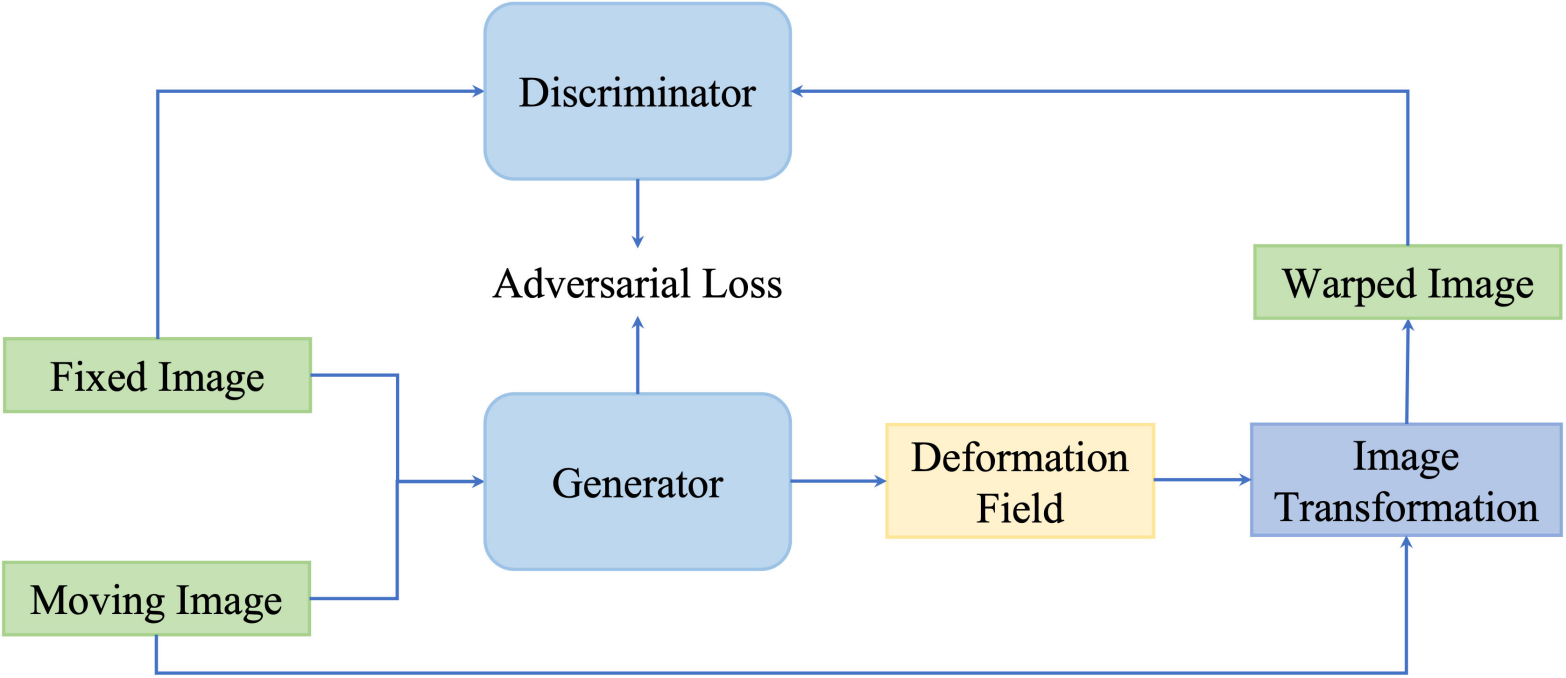
A general pipeline of GAN-based deformable image registration methods. This network is optimized by optimizing the adversarial loss function.

Although similarity-based unsupervised methods are not trained with existing registration transformations, analyzing transformations in terms of spatially corresponding patches could be challenging in multimodal registration and can negatively impact registration results. For overcoming issues existing in multimodality translation, generative adversarial network (GAN)-based unsupervised framework performs the training process in an adversarial setting, in which a discriminator predicts the probability that the generation of new images will match the distribution of the input training data. **Figure 7** is a general framework of GAN-based deformable image registration methods.

Mahapatra et al. (116) utilized both cycGAN and cGAN to predict both warped images and DVF. cGANs are used for multimodal registration so that the generated output image (the moving image after transformation) is similar to the original (based on intensity distribution) while having the same landmark locations as the reference image (from a different modality). The loss function for image generation is modified to incorporate adversarial loss and cycle consistency loss to obtain consistent and realistic deformation fields, which allow new image pairs from the untraining set to be registered. This method outperformed conventional retinal image registration with MAD, MSE, and Hausdorff distance.

The GAN framework has shown promising results in cross-modality medical image registration. Not only addressing multimodal registration, currently, more papers implement GAN in diverse registration tasks. Neel et al. claimed a GAN approach to construct the conditional deformable template across datasets from different populations. Despite GAN showing great potential in its generative and discriminative features, the validation of output warped image accuracy is still required to be investigated, especially in unpaired images with minor abnormality regions.

### 4.4 Weakly supervised methods

**Figure 8:**
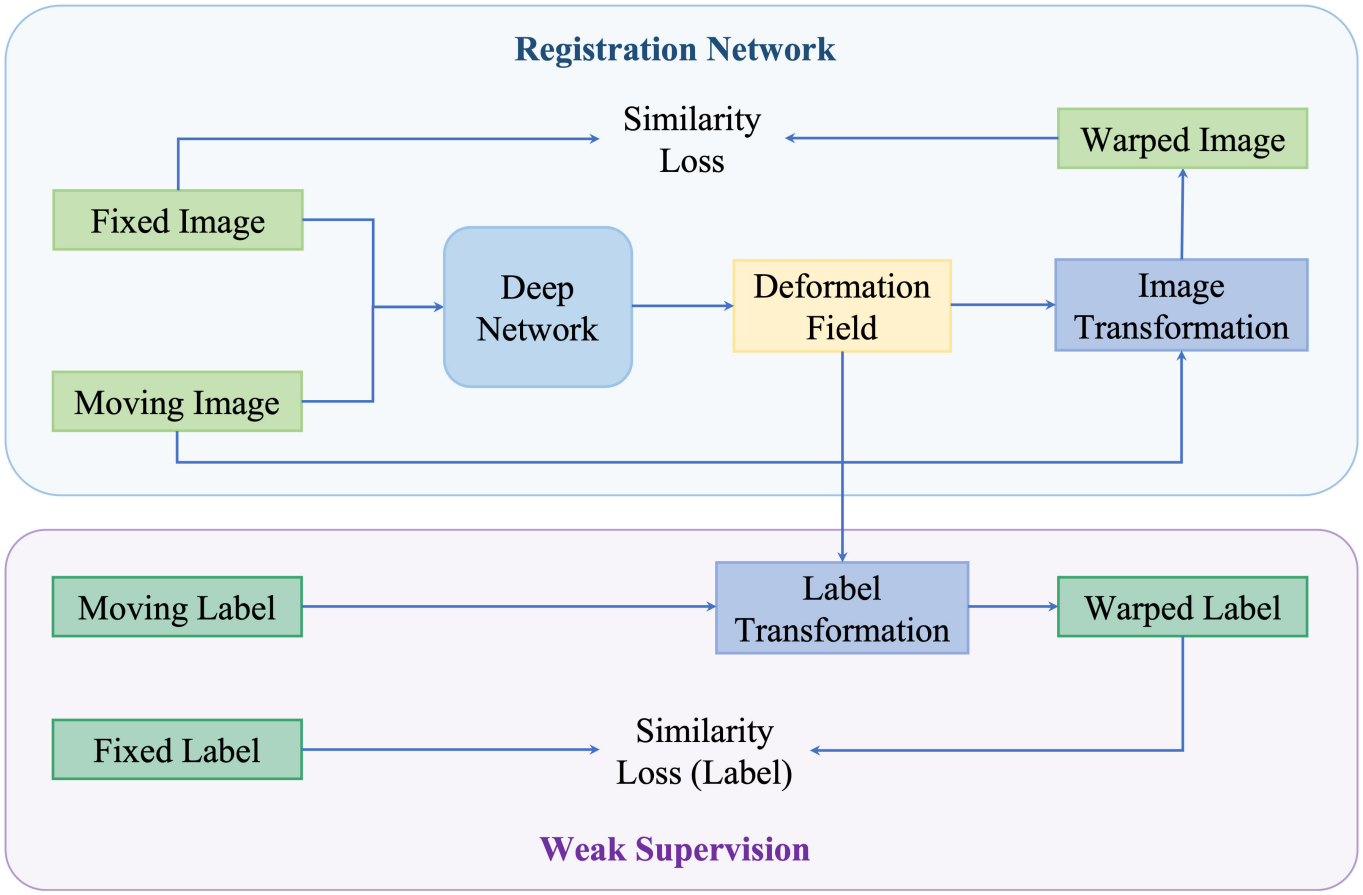
An illustration of weakly supervised deformable registration methods. The weak supervision is achieved by adding anatomical labels in the network.

Neither using real or synthetic deformation vector field as supervision nor directly predicting deformation field without supervision, weakly supervised methods use labels as supervision, for example, segmentations and key points; such anatomical labels are feasible in practice and credible for training. **Figure 8** is a general framework of weakly supervised deformable image registration methods. Generally, these networks are trained by minimizing anatomical losses, which ensures that the predicted segmentations match the anatomical labels and provide anatomy consistency monitoring. Weakly supervised methods naturally improve the performance of registration by introducing the anatomical constraints. A conclusion of weakly supervised methods is presented in **Table 5**.

**Table 5:** Weakly supervised medical image registration methods.

Reference	Supervision	Organ	Modality	Dimension	Model	Evaluation	Source
(128)	Segmentations	Lung	X-ray	2D	GAN	DSC, HD, TRE	MLMI 2018
(129)	Anatomical labels	Prostate	MRI-US	3D	CNN	DSC, TRE	MIA 2018
(130)	Anatomical labels	Prostate	MRI-US	3D	CNN	DSC	ISBI 2018
(131)	Biomechanical Simulations	Prostate	MRI-US	3D	GAN	DSC, TRE	MICCAI 2018
(132)	Anatomical labels	Cardiac	MRI	3D	CNN	DSC	BFM 2019
(133)	Anatomical labels	Brain	MRI	3D	CNN	DSC	MLMI 2019
(134)	Anatomical labels	Brain	MRI	3D	CNN	DSC	MICCAI 2019
(135)	Segmentations	Brain, Knee	MRI	3D	CNN	DSC	MICCAI 2019
(136)	Contours	Lung	CT	3D	CNN	DSC	MICCAI 2019
(137)	Key points	Lung	CT	3D	CNN	DSC	MIDL 2019
(94)	Segmentations	Brain	MRI	3D	U-Net	DSC	TMI 2019
(138)	Anatomical labels	Brain	MRI	3D	CNN	DSC, ASD	MICCAI 2019
(125)	Noisy labels	Lung	CT	3D	U-Net	DSC, HD, STD	MICCAI 2020
(139)	Segmentations	Brain	MRI	3D	CNN	DSC, HD, ASSD	Med Phys 2021
(140)	Lung masks and key points	Lung	CT	3D	CNN	TRE, DSC, ASD	MIA 2021
(141)	Key points	Lung	CT	3D	MLP	TRE	MIDL 2021
(127)	Cancer labels	Prostate	Histopathology, MRI	2D, 3D	CNN	Landmark error, DSC, HD	MICCAI 2021
(142)	Boundaries	Cardiac	MRI	3D	CNN	DSC, HD	MICCAI 2021
(143)	Boundaries	Liver	CT-Laparoscopic	3D-2D	CNN	TRE	MICCAI 2021

CT, computed tomography; MRI, magnetic resonance imaging; CNN, convolutional neural network; MLP, multi-layer perceptron; GAN, generative adversarial network; DSC, Dice coefficient; ASSD, average symmetric surface distance; TRE, target registration error; ASD, average surface distance; HD, Hausdorff distance; STD, standard deviation.

Hu et al. (129) presented an end-to-end convolutional neural network to inference deformation field for 3D MRI and US image registration. Image pairs with multiple labeled corresponding structures were used for the training, and only unlabeled image pairs are used for testing. Xu et al. (135) proposed DeepAtlas, a network for weakly supervised registration and semi-supervised segmentation. The networks were trained jointly through an anatomy similarity loss that penalized the difference between the transformed segmentation of the moving image and the target image’s segmentation. Estienne et al. (125) developed a registration network for abdominal CT registration by applying spatial gradients and noisy segmentation labels. Recently, Hering et al. (140) proposed a multi-level framework for lung CT image registration by introducing the constraints of lung masks and key points to make the airways and arteries more aligned.

Furthermore, image registration and image segmentation are inextricably linked and can help one another. On one hand, labeled atlas images can be utilized for segmentation through image registration. On the other hand, segmentations are capable of adding extra anatomical constraints for image registration, and segmentations are also useful in evaluating the registration algorithms. Therefore, this weakly supervised paradigm is applicable in medical image segmentation, such as the DeepAtlas described in (135) and the method (144).

### 4.5 Latest methods/recent directions

The latest deformable medical image registration algorithms are listed in **Table 6**; these methods adopted recent deep learning techniques, such as Transformer (160), contrastive learning (161), meta learning (162), neural ODE (163), and the diffusion model (164).

**Table 6:** Image registration with latest techniques.

Reference	Technique	Organ	Modality	Dimension	Evaluation	Source
(145)	Transformer	Brain	MRI	3D	DSC	MICCAI 2021
(146)	Attention	Prostate	MRI-US	3D	SRE	MICCAI 2021
(147)	Uncoupled learning	Brain	MRI	3D	DSC	MICCAI 2021
(148)	Meta learning	Brain	MRI	3D	Distance, NCC	arXiv 2021
(149)	Transformer	Brain	MRI	3D	DSC	arXiv 2021
(150)	Transformer	Brain	MRI	3D	DSC, [*J*]	MIA 2022
(151)	Contrastive learning	Brain	MRI	3D	DSC	arXiv 2020
(152)	Contrastive learning	Brain	MRI	3D	Landmark error	SSMI 2021
(153)	Transformer	Cardiac	CT	3D	DSC, ASD HD	MIA 2022
(154)	Transformer	Torso	PET-CT	2D-3D	IOU, CPE	IMIP 2022
(155)	Knowledge distillation	Liver, Brain	CT, MRI	3D	DSC, [*J*]	TMI 2022
(104)	Neural ODE	Brain	MRI	3D	DSC, RMSE	MICCAI 2021
(156)	Neural ODE	Brain	MRI	3D	DSC	CVPR 2022
(157)	Diffusion model	Brain	MRI	3D	DSC	arXiv 2021
(158)	Transformer	Brain	MRI	3D	Dice	MICCAI 2022
(159)	Transformer	Heart	CT	2D	Dice, [*J*]	MICCAI 2022

CT, computed tomography; US, ultrasound; MRI, magnetic resonance imaging; PET, positron emission tomography; RMSE, root mean square error; SRE, surface registration error; NCC, normalized cross-correlation; ASD, average surface distance; DSC, Dice coefficient; HD, Hausdorff distance; [J], Jacobian determinant; IOU, intersection over union; CPE, center position error.

Transformer (160) is the most popular technique in medical image registration. Chen et al. (149) designed a ViT-V-Net for brain MRI image registration. They adopted a hybrid ConvNet-and-Transformer architecture to apply ViT for high-level feature learning. Their experimental results proved that simply replacing the network backbone of VoxelMorph by Vit-V-Net could improve the performance. They also extended the ViT-V-Net and presented TransMorph (150), a hybrid Transformer-ConvNet framework. In this framework, they employed the Swin Transformer (165) as the encoder to learn the spatial transformation between the input images. Then, a decoder constructed with ConvNet processed the features from the Transformer encoder and exported the dense deformation field. To provide a smooth and topology-preserving deformation field, they also presented diffeomorphic variations of TransMorph.

For contrastive learning (161), except for the application in brain MRI registration, Jiang et al. (166) developed a network to generate pseudo-CT from MRI for brain radiotherapy based on a contrastive unpaired translation network (CUT) (167). Comparing to GAN-based generation methods, their network can capture more structure and texture information that is useful to generate more realistic CT images.

Neural ODE (163) is developed to depict more complex dynamic systems. Compared to well-known deep learning models like ResNet and U-Net, neural ODE models are more efficient in terms of memory and parameters. Neural ODE models have the advantage of adaptive computing, making them potentially appropriate for application in medical applications. Additionally, the dynamics of optimization are naturally continuous. These benefits encourage researchers to study how to use neural ODEs to optimize the registration of medical images. Xu et al. (104) proposed to formulate the traditional optimization strategy in registration methods as a continuous mechanism and learn the optimizer through a multi-scale neural ODE model. Wu et al. (156) presented a novel and accurate diffeomorphic image registration framework using Neural ODES and explored the potential of combining the advantages of neural networks and flow formulations. In this work, every voxel was portrayed as a moving particle, and the entire collection of voxels was regarded as a high-dimensional dynamical system, with each voxel’s trajectory determining the appropriate deformation field.

## 5 Discussion

From 2013, researchers began to apply deep learning techniques to image registration, and the applications of deep models for registration flourished from 2017. Deep learning-based methods have shown high computational efficiency and comparable accuracy compared with traditional methods. Deep similarity-based models and reinforcement learning-based methods adopt iterative strategy, which is time-consuming. Supervised methods need ground truth supervision that is impractical to obtain. In contrast, unsupervised methods and weakly supervised methods are less reliant on ground truth information and become hot topics for deep learning-based registration algorithms. Recently, popular networks, such as Transformer and contrastive learning, are explored for deformable medical image registration and achieved promising results.

Complexity is an important factor that needs to be considered when designing the registration networks. Parameters and floating point operations (FLOPs) are common criteria used to measure the complexity of the model. In view of the fact that the calculation of FLOPs involves the size of input images, we here discuss the model complexity in terms of parameters. Due to the large number of variants of network architecture, we provide approximate model parameters of the backbone networks that are commonly used for deformable registration in **Table 7**. We can see that GAN has the smallest model parameter and the parameter of Transformer expands significantly. Expect the backbone parameters, additional modules and branches also add the complexity of the registration models.

**Table 7:** Parameters of the networks used for deformable image registration.

Network	GAN	CNN	U-Net	MLP	Transformer
**Parameters**	11M	14M	19M	15M	46M

In conjunction with the previous discussion about complexity, here we discuss how to choose a registration network for a specific task from different categories. Extra label is the first factor that needs to be considered; if the label is available, then the semi-supervised methods are preferable and have the potential for high registration accuracy. The second factor for choosing a network is the data size and GPU memory; the Transformer is unavailable when the data size is large and GPU memory is limited. For multimodality registration, GAN-based methods are suitable as GAN can ensure that the generated registered image has the same characteristic (intensity distribution) as the source image while being similar to the target image in terms of structures. The supervised methods are applicable when the ground truth deformation vector fields are provided or they are easy to obtain. Lastly, the unsupervised similarity-based methods have become more popular in recent years, and they are appropriate in many deformable registration tasks as they do not require extra information.

Here, we discuss the remaining issues that need to be studied and potential directions for future research on deformable image registration:

1. Registration Models. CNNs, SAEs, GANs, DRLs, and deep RNNs make up the majority of the recently developed deep learning algorithms used for medical image registration, whereas other models also offer a significant potential for advancement. In contrast to the medical image areas, we think that the majority of future trends and contributions will originate from other subjects, such as computer vision and machine learning. Nowadays, the popular neural ODE and diffusion models have also been explored for deformable image registration. In the future, models in other areas may also have the potential to be used for deformable medical image registration.
2. Diffeomorphic Registration. Due to characteristics like topology preservation and transformation invertibility, diffeomorphic image registration using deep models has caught the attention of researchers. In deep learning-based deformable registration methods, two main strategies are proposed to guarantee the diffeomorphism of the deformation fields. The first strategy is to add an explicit constraint (regularization) for the learned deformation field. Usually, the constraints are performed by penalizing the small and large values of the Jacobian determinant. The other method is to introduce diffeomorphic integration layers. The integration is performed by scaling and squaring the stationary velocity field. However, there are still some limitations, and the guarantee for diffeomorphism in learning-based methods remains a challenging problem. Therefore, developing deformable registration networks that can guarantee the diffeomorphism of the deformation field is a prospective research topic.
3. Registration Efficiency. From the point of view of registration efficiency, the training time and network parameters still need promotion, especially for 3D image registration. For example, when training 3D lung CT registration network, a GPU card with a memory larger than 12 G is necessary. Thus, a lightweight network for 3D image registration is also a potential research direction. Considering the applications in computer-assisted surgeries, a shorter training time is preferable, so networks that can converge rapidly are appealing.
4. Large Deformation of Soft Tissues. The learning of large and irregular deformation caused by organ movement (e.g., lung respiratory motion) is still a challenging problem and needs further research. In learning-based registration methods, one strategy to address the large deformation is to adopt multi-stage coarse-to-fine architecture. This kind of method consumes large GPU memory and the training is time-consuming as the networks are trained separately. Another method for learning large deformation is to construct cascaded networks, in which each cascaded network learns an intermediate deformation field, and the source image is recursively and progressively warped by the field, finally aligned to the target image. This kind of network is still memory-consuming and is difficult to converge as all cascaded networks are trained simultaneously. Therefore, learning of large deformation remains an outstanding issue and efforts are needed.
5. Registration Constraints. Predicting the deformation field without constraints can lead to warped moving image with distorted unrealistic organ appearances. The most commonly used technique for tackling this issue is to add the L2 norm on the gradient of the deformation field to regularize the predicted deformation. The magnitude of the field might be restricted by the employment of such regularization terms. Considering this, adding anatomical constraints to deep networks can help to generate realistic deformations. More importantly, applying appropriate constraints can help the network learn a deformation field that keeps the topology of the input image pairs so that the registration is more reliable in clinical applications. Thus, exploring constraints for particular tasks is another attractive research point.

## 6 Conclusion

We provide a comprehensive survey for the development of deep learning-based medical image registration methods in this article. We also have a thorough analysis of publicly available datasets as well as their details in order to assist algorithm benchmarking and future studies. The evolution of learning-based image registration algorithms has followed a similar path to that of the deep learning models. Image registration neural networks are gradually moving from handling 2D images to 3D or 4D (dynamic) volumes, and converting from supervised methods to unsupervised methods and weakly supervised methods. Recent advances are also reviewed, including those methods that adopt Transformer, contrastive learning, and other latest techniques. We also present the statistical analysis of our selected papers from the aspects of modalities, organs, evaluation metrics, and supervision. Future research challenges and directions are also discussed, including how to speed up registration in higher dimensions, reduce the requirement for ground truth during training, and use anatomical constraints to produce deformation fields that are more realistic while retaining anatomical consistency.

## Author contributions

JZ and BG conducted literature research and analysis. The introduction, methods, discussion, and conclusion parts of this manuscript were written by JZ. The statistical analysis, datasets, and GAN-based methods were written by BG. YS and JQ revised the paper. All authors contributed to the article and approved the submitted version.
